# Preparation and Properties of A Hyperbranch-Structured Polyamine adsorbent for Carbon Dioxide Capture

**DOI:** 10.1038/s41598-017-04329-w

**Published:** 2017-06-20

**Authors:** Hui He, Yajie Hu, Shuixia Chen, Linzhou Zhuang, Beibei Ma, Qinghua Wu

**Affiliations:** 1 PCFM Lab, School of Chemistry, Sun Yat-Sen University, Guangzhou, 510275 P.R. China; 2 Materials Science Institute, Sun Yat-Sen University, Guangzhou, 510275 P.R. China; 3 College of Light Industry and Food Engineering, Guangxi University, Nanning, 530004 P.R. China

## Abstract

A fibrous adsorbent with amino-terminated hyperbranch structure (PP-AM-HBP-NH_2_) was prepared by grafting hyperbranched polyamine (HBP-NH_2_) onto the acrylamide-modified polypropylene (PP) fibers. The grafting of AM on PP fibers provided the active sites for introducing HBP-NH_2_ onto the PP fibers. This kind of “grafting to” procedure to synthesize hyperbranch-structured fiber could overcome the disadvantages of stepwise growth procedure, avoiding the complicated synthesis process and the requirement of strict experimental conditions. The grafted HBP-NH_2_ was three-dimensional dentritic architecture and had a large number of pores existing within the grafted polymers, which is favorable for CO_2_ molecules to diffuse into the HBP-NH_2_. Therefore, the as-prepared PP-AM-HBP-NH_2_ fibers showed a high adsorption capacity (5.64 mmol/g) for CO_2_ in the presence of water at 25 °C, and the utilization efficiency of alkyl amino groups could reach 88.2%, demonstrating that the hyperbranched structure of adsorbents can greatly promote adsorption capacity and efficiency. This could be attributed to better swelling properties and lower mass transfer resistance to CO_2_ of the hyperbranched adsorbent. PP-AM-HBP-NH_2_ also showed excellent regeneration performance, and it could maintain the same adsorption capacity for CO_2_ after 15 recycle numbers as the fresh adsorbent.

## Introduction

Great energy demand intensifies the combustion of fossil fuels (coal, petroleum and natural gas), resulting in escalated global CO_2_ emission, which is one of the major causes of global warming. One efficient way to control atmospheric CO_2_ concentration is to reduce CO_2_ emission from flue gas which takes up 86% of anthropogenic greenhouse gas^1^. The two main methods to capture CO_2_ from the flue gas are physical or chemical absorption and adsorption. Though absorption, using aqueous solvents, has many advantages, such as higher selectivity and absorption capacity for CO_2_ removal from flue gas, it has drawbacks like high volatility leading to absorbent consumption, high alkalinity causing equipment erosion and high energy consumption for regeneration^2^. In contrast, solid adsorbents overcome these disadvantages and exhibit promising application in carbon capture.

Among solid adsorbents, solid amine adsorbents such as amino-functionalized MCM-41^3^, SBA-15^4, 5^, Zeolite 13X^6, 7^ and activated carbon^8, 9^ have been used, having attracted much attention due to their physical and chemical adsorption of CO_2_. Solid amine adsorbents have advantages such as lower energy consumption for regeneration, higher adsorption capacity and efficiency, higher stability and less erosion for equipments. However, large amount of amine loading on these porous supports, particularly microporous and mesoporous silica supports may cause serious pore blockage and CO_2_ diffusion resistance, leading to the reduction of adsorption capacity for CO_2_^10^. Thus, it is necessary to choose another support instead of porous supports to retain the adsorption capacity while increasing the loads of amino.

Nowadays, Fibrous adsorbents have gained much attention due to their light weight, great mechanical property, large external surface area, short transit distance, low pressure drops, and flexibility. Lots of works about fibrous adsorbents have done in our group^10–14^. Li^11^ chose epichlorohydrin as cross-linking reagent to graft PEI onto glass fiber (GF) and prepared GF-PEI adsorption fiber with an adsorption amount of 4.12 mmol/g. Yang^12^ adopted (NH_4_)_2_S_2_O_8_/NaHSO_3_ redox as initiator reagent to graft allylamine onto polyacrylonitrile (PAN) and gained PAN-AF fiber with an adsorption amount of 6.22 mmol/g when the grafting degree was 60.0 wt%. These fibers all have great regeneration abilities. Besides the substrate, the amino content and the structure of amino of the adsorbents are the important factors that affect the CO_2_ adsorption performance of the adsorbents. Xu^15^ has developed a new procedure that is effective and safe to synthesize hyperbranch-structured fiber by stepwise growth using N-(2-chloroethyl)-benzaldimine as monomer. The maximum adsorption capacity of hyperbranched solid amine fiber reaches 5.53 mmol/g at 30 °C. These results demonstrate that hyperbranched structure can significantly increase the adsorption capacity and efficiency. However, this kind of stepwise growth procedure is complicated and requires strict experimental conditions because of its multiple steps.

Amino-terminated hyperbranched polyamine is easier to prepare compared with dendritic molecules, because it doesn’t require uniform structure and high symmetry^16–23^. Moreover, there are abundant amino groups which can be the adsorption sites of CO_2_ in the hyperbranched polyamine. Using hyperbranched polyamine as animation reagent is a promising way to prepare hyperbranch-structured adsorbents with high CO_2_ adsorption capacity.

**Figure 1:**
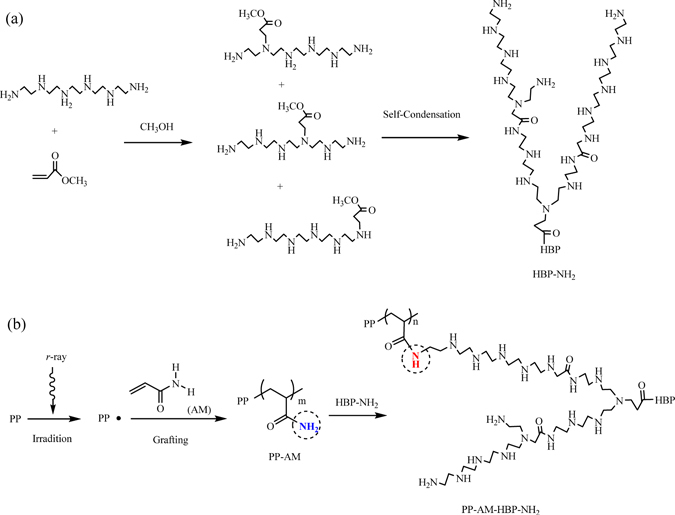
Reaction scheme of HBP-NH_2_ preparation (**a**) and PP-AM-HBP-NH_2_ preparation (**b**).

**Figure 2:**
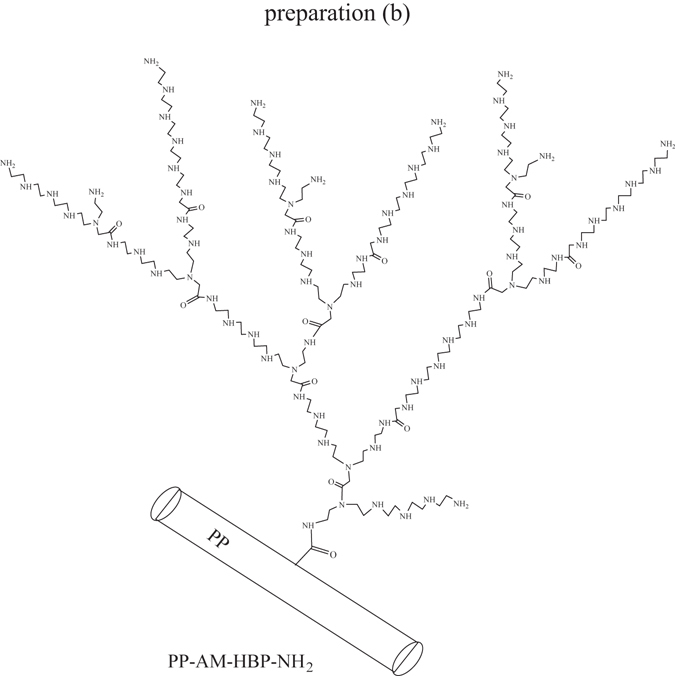
A possible structure of PP-AM-HBP-NH_2_.

In this study, a simple procedure has been developed for the synthesis of hyperbranch-structured fibers, in which the amino-terminated hyperbranched polyamine (HBP-NH_2_) was first prepared through Michael addition reaction of pentaethylenehexamine (PEHA) and methyl acrylate (MA), and then grafted onto acrylamide (AM)-modified polypropylene (PP) fibers (Figs 1 and 2). The chemical structure, adsorption property and regeneration ability of the PP based amino-terminated hyperbranch-structured fiber (PP-AM-HBP-NH_2_) were evaluated in detail.

## Results and Discussion

### Control of amino density of PP-AM-HBP-NH_2_ adsorbent

The HBP-NH_2_ with high amino density was prepared through Michael addition reaction of PEHA and MA. The molecular weight and amino content of the HBP-NH_2_ were listed in Table 1.

**Table 1:** Molecular weight and amino content of HBP-NH_2_ where the amide content (mmol/g) of HBP-NH_2_ was calculated from the oxygen mole content, alkyl amino content of HBP-NH_2_ was calculated by subtracting the amide content from total amino amount.

Mw (g/mol)	C (wt%)	N (wt%)	H (wt%)	O (wt%)	Amino amount (mmol/g)	Amide content (mmol/g)	Alkyl amino content (mmol/g)
10820	50.98	30.29	12.93	5.80	21.64	3.63	18.01

As shown in Table 1, molecular weight and nitrogen content of HBP-NH_2_ were 10820 g/mol and 30.29 wt%, respectively, and the alkyl amino content could reach 18.01 mmol/g.

**Figure 3:**
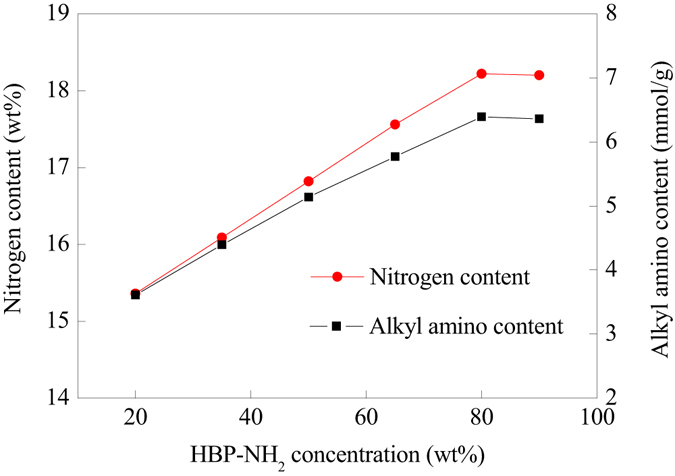
Nitrogen content and alkyl amino content of PP-AM-HBP-NH_2_ prepared in various concentration of HBP-NH_2_.

HBP-NH_2_ was combined onto AM-grafted PP fiber (PP-AM) through the reaction of amide on PP-AM and amino groups on HBP-NH_2_. As shown in Fig. 3, with the increase of HBP-NH_2_ concentration in the synthesis system, more and more HBP-NH_2_ was combined onto PP fiber, thus, the nitrogen content and alkyl amino content of the prepared PP-AM-HBP-NH_2_ increased. When the concentration of HBP-NH_2_ was 80%, the nitrogen content could reach to 18.45 wt%. Whereafter, the nitrogen content and alkyl amino content didn’t increase with the further increase of the HBP-NH_2_ concentration. Increasing HBP-NH_2_ concentration would provide more amino groups that could be grafted onto PP-AM fibers, however, when HBP-NH_2_ concentration exceed a certain value (80 wt% in this case), viscosity of the reaction system increased and the mobility decreased, which would reduce diffusion and mass transfer of the HBP-NH_2_ molecules, thus decrease the amount of grafted HBP-NH_2_.

**Figure 4:**
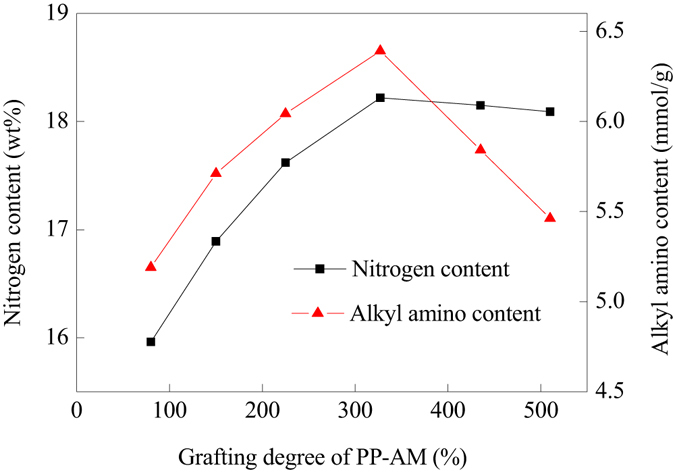
Nitrogen content and alkyl amino content of PP-AM-HBP-NH_2_ prepared by using PP-AM with various grafting degree.

In order to provide reactive sites to combine HBP-NH_2_, AM was firstly grafted onto PP fiber by simultaneous grafting copolymerization. The effect of the grafting degree of AM on the amino density of the prepared PP-AM-HBP-NH_2_ was examined. As shown in Fig. 4, the nitrogen content of PP-AM-HBP-NH_2_ increased with the grafting degree of PP-AM till the grafting degree was 320%. Accordingly, the alkyl amino content of PP-AM-HBP-NH_2_ also increase with grafting degree of PP-AM, however, it decreased after the grafting degree of PP-AM was 320%. The nitrogen content and alkyl amino content reached their maximum values (18.22 wt%, 6.39 mmol/g, respectively.) when the grafting degree of PP-AM was 320%. As we know, amount of amide groups on PP-AM increased in proportion to its grafting degree, amide groups could react with HBP-NH_2_ by amide subsitution reaction, thus the more the amide groups, the higher the nitrogen content and alkyl amino content. However, with the increase of grafting degree of PP-AM, the grafting layer on the surface of PP fibers became thicker, which was not beneficial for HBP-NH_2_ molecules to diffuse into the internal layer and react with the amide groups inside. Therefore, nitrogen content didn’t further increase with the grafting degree, and the proportion of alkyl amino content would decrease due to the unreacted amide.

### Morphology and Chemical characterization

**Figure 5:**
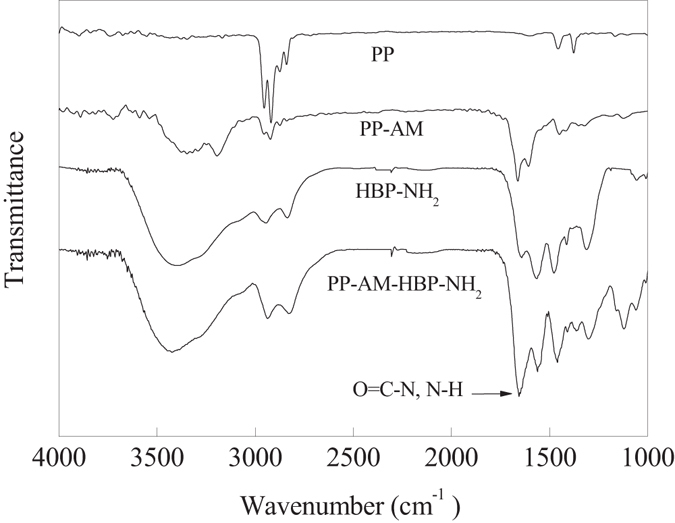
FT-IR spectra of PP, PP-AM, HBP-NH_2_ and PP-AM-HBP-NH_2_.

Figure 5 showed FT-IR spectra of PP, PP-AM, and PP-AM-HBP-NH_2_, respectively. Compared with the spectra of PP fibers, the PP-AM fibers were characterized by the peaks at 1653 cm^−1^ and 1606 cm^−1^ corresponding to the stretching vibrations of C=O and −NH, which proved the existence of amide bond. Two broad adsorption peaks at 3000–3500 cm^−1^, which are attributed to primary amino, were observed. The existence of amide bonds and primary amino confirmed that AM had successfully grafted on PP fibers. The absorption peaks at 1573 cm^−1^, 1460 cm^−1^, and 1116 cm^−1^ in the spectrum of HBP-NH_2_ are corresponding to the bending of amino groups (N-H) in HBP-NH_2_. The wide absorption peak at 3000–3500 cm^−1^ in the spectrum of PP-AM-HBP-NH_2_ is corresponding to the N-H bond of primary amino; the absorption peaks at 2820 cm^−1^ and 2928 cm^−1^ are corresponding to symmetric and asymmetric stretching vibrations of −CH_2_−, respectively. The peaks at 1653 cm^−1^ and 1573 cm^−1^ are assigned to the stretching vibrations of C=O and N-H, respectively, which represented the existence of amide bond. The absorption peaks at 1573 cm^−1^, 1460 cm^−1^, and 1116 cm^−1^ are corresponding to the bending of amino groups (N-H) in PP-AM-HBP-NH_2_. These results testified the substituting of HBP-NH_2_ for amino groups in amide.

**Figure 6:**
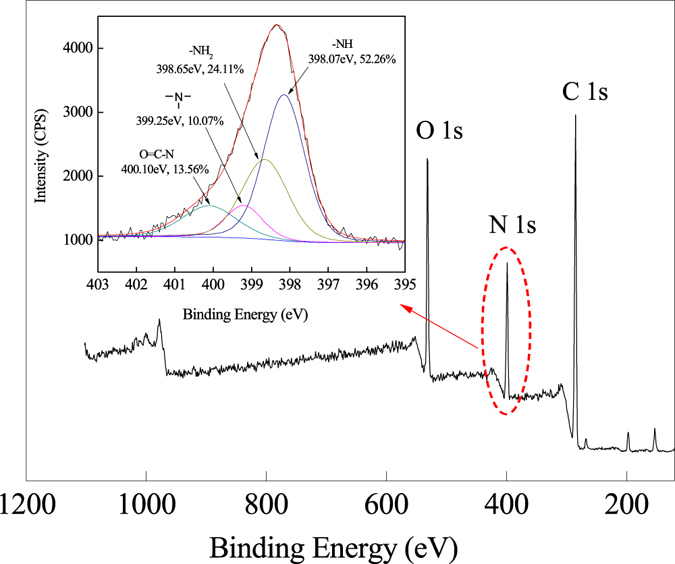
N1s peak processing of XPS of PP-AM-HBP-NH_2_.

The nitrogen-containing groups were further resolved into amide and alkyl amino (including 1, 2, 3° amino) by N1s peak processing of XPS spectrum, which were showed in Fig. 6. It could be resolved into four peaks at 400.10 eV (13.56%), 399.25 eV (10.07%), 398.65 eV (24.11%), and 398.07 eV (52.26%), these peaks were the characteristics of amide, tertiary amino, primary amino, and secondary amino, respectively. The presence of tertiary amino indicated that the HBP-NH_2_ was grafted onto the PP-AM, and the target hyperbranch-structured fibers were successfully prepared.

**Figure 7:**
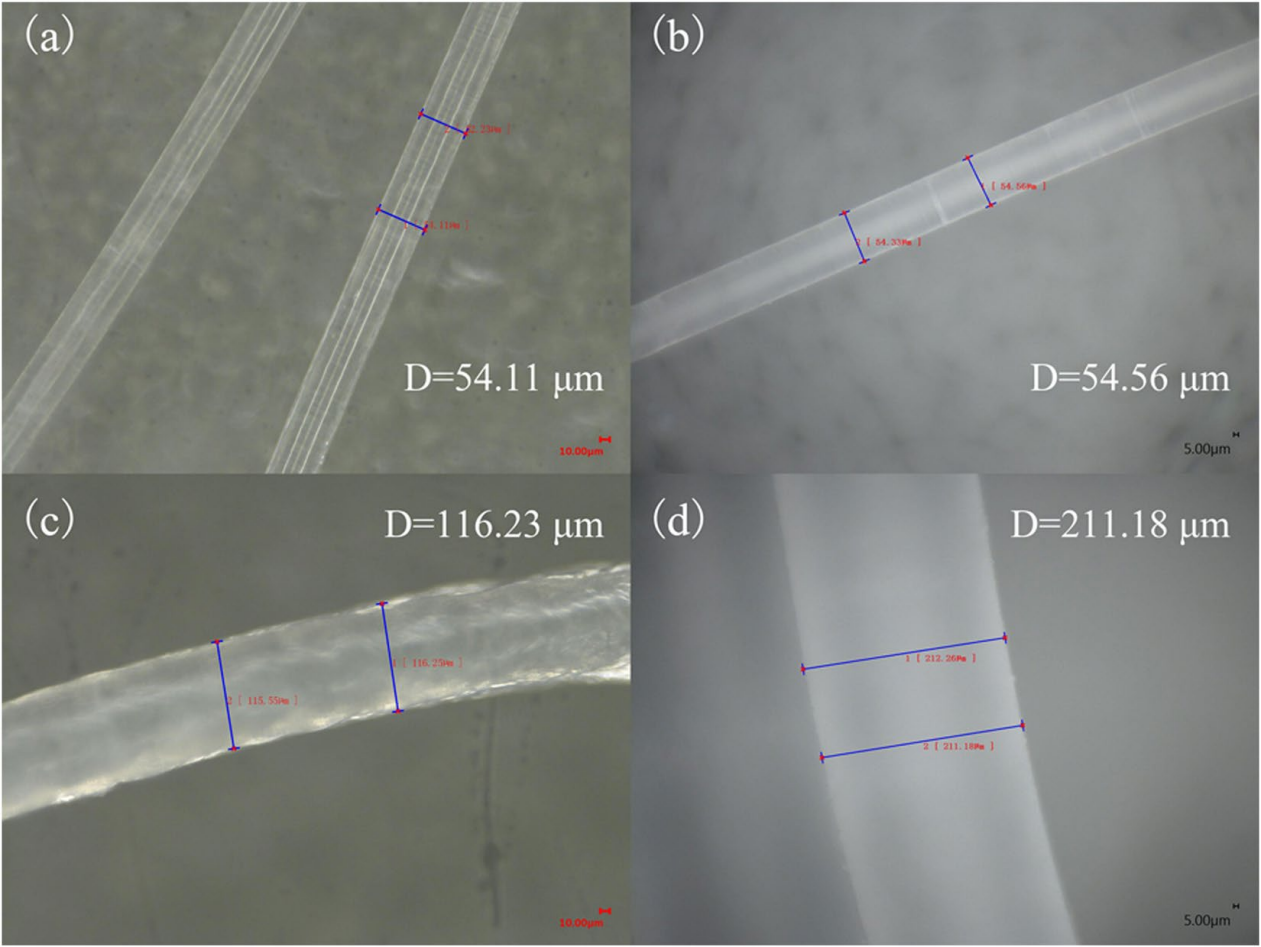
Ultra-depth three-dimensional microscope images of PP (**a**,**b**) and PP-AM-HBP-NH_2_ (**c**,**d**) under dry (left) and moisture (right) conditions.

During the grafting of AM onto PP fibers, AM copolymerized with PP to form PP-AM copolymers with polyamide oligomer as side chains^24^, the diameter of the PP-AM thus was increased to 95.52 μm from 54.11 μm (PP). And then its diameter further increased to 116.23 μm after grafting HBP-NH_2_ onto PP-AM (Figure S1 of the supporting information). As shown in Fig. 7, no significant changes in diameter of PP fiber were observed after swelling in water at 25 °C, the swelling degree was only 0.8% due to the hydrophobicity of PP fiber. However, under moisture condition, PP-AM-HBP-NH_2_ could present good swelling property, its diameter increased from 116.23 μm under dry condition to 211.18 μm in the presence of water, the swelling degree was 81.69% in the presence of water at 25 °C, which would be beneficial to the diffusion of CO_2_ into the internal layer.

### Adsorption property of PP-AM-HBP-NH_2_

#### Effect of alkyl amino content

**Figure 8:**
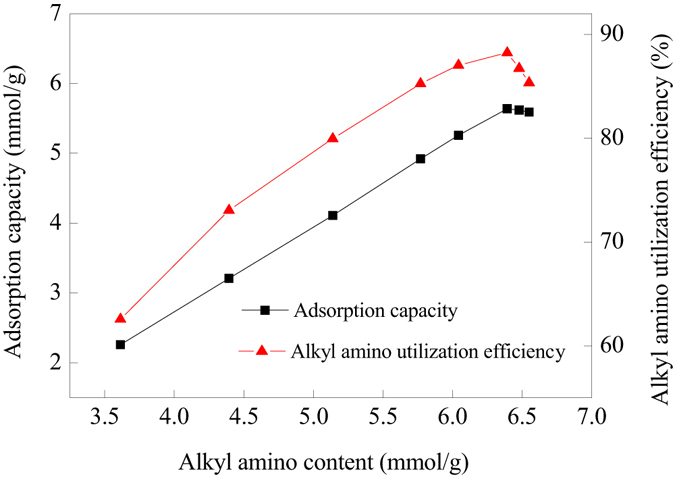
Effects of alkyl amino content on adsorption capacity of PP-AM-HBP-NH_2_.

**Figure 9:**
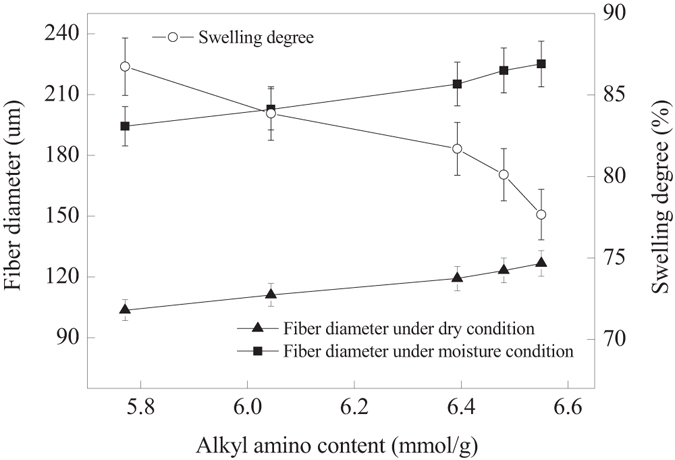
Diameter and swelling degree of PP-AM-HBP-NH_2_.

As shown in Fig. 8, The CO_2_ adsorption capacities and alkyl amino utilization efficiencies almost linearly increased with alkyl amino content. However, when HBP-NH_2_ grafting layer became thicker and thicker film-diffusion resistance became the key factor affecting the adsorption kinetics of CO_2_. Moreover, the number of the accessible adsorption groups would be reduced when the grafting layer of HBP-NH_2_ was too thicker. It was further verified by swelling property analysis (Fig. 9), the diameters of PP-AM-HBP-NH_2_ in both dry and moisture conditions increased with its alkyl amino content, but the swelling degree showed a decrease tendency. When alkyl amino content exceed 6.39 mmol/g, the swelling degree decreased sharply, which would greatly limit diffusion of CO_2_ into the internal layer, thus lead to the decrease of the adsorption capacity and alkyl amino utilization efficiency.

#### Effect of adsorption temperature

**Figure 10:**
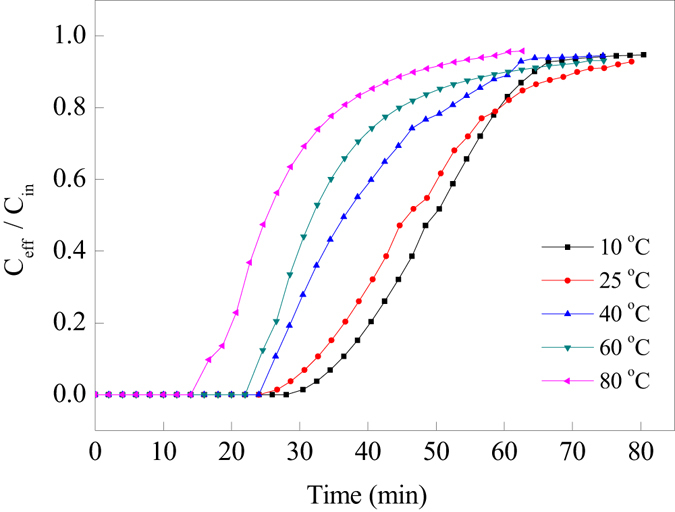
Breakthrough curves of CO_2_ adsorption on PP-AM-HBP-NH_2_ at different adsorption temperatures (alkyl amino content of PP-AM-HBP-NH_2_ was 6.39 mmol/g).

The results in Fig. 10 indicated that CO_2_ could be completely adsorbed by PP-AM-HBP-NH_2_ adsorbent at the early stage. It lasted for longer time for the complete adsorption of CO_2_ under low temperature. Increasing adsorption temperature would accelerate the breakthrough of CO_2_ from the adsorption bed. Table 2 showed that the adsorption capacity was higher at lower adsorption temperature. The CO_2_ adsorption capacity of PP-AM-HBP-NH_2_ at 10 °C could reach 5.90 mmol/g, which was two times as much as that at 80 °C (2.82 mmol/g). Low temperature was beneficial to the adsorption of CO_2_ on solid amine adsorbent because it was an exothermic process.

**Table 2:** Adsorption capacities and alkyl amino utilization efficiency of PP-AM-HBP-NH_2_ fibers (alkyl amino content was 6.39 mmol/g) at different adsorption temperatures.

Temperature (°C)	Adsorption capacity (mmol/g)	Alkyl amino utilization efficiency (%)
10	5.90	92.3
25	5.64	88.2
40	4.80	75.1
60	3.81	59.6
80	2.82	44.1

Unlike the linear amination reagents, HBP-NH_2_ possess three-dimensional dentritic architecture and a large number of pores exist within the grafted oligomers^10, 11, 13, 14^, the average diameter of pore is 0.5 nm^16^ (greater than that of CO_2_ molecular diameter (0.33 nm)). This kind of pores is favorable for CO_2_ molecules to diffuse into the internal layer and react with the amino groups inside, and therefore greatly promote the adsorption capacity and amino utilization efficiency (Table 2). Moreover, the single branched molecule chain of HBP-NH_2_ would provide various amino adsorption sites for CO_2_ capture, and the amino groups can fully contact with CO_2_ molecules. Thus alkyl amino utilization efficiency of PP-AM-HBP-NH_2_ could reach 88.2% at 25 °C, which was much higher than that of the other linear amine reagents (Table 3).

**Table 3:** Comparison of CO_2_ adsorption capacity of amine functionalized fibrous sorbents (adsorption temperature: 25 °C, CO_2_ concentration: 10%).

Substrate*	Grafted monomer**	Amine	Adsorption capacity (mmol/g)	Amino utilization efficiency (%)	Ref.
PP	AM	HBP-NH_2_	5.64	88.2	This work
PP	AM	PEI	5.91	56.0	10
GF	ECH	PEI	4.12	57.0	11
PP	GMA	TETA	4.72	46.1	13
VF	AM	TEPA	1.92	40.4	14
VF	AM	EDA	2.82	60.1	14

*GF: glass fibers, VF: viscose fibers, ** ECH: epichlorohydrin, GMA: glycidyl methacrylate.

Compared with other fibrous adsorbents reported (Table 3), PP-AM-HBP-NH_2_ adsorption fibers showed relatively high adsorption efficiency. More interestingly, Wu^10^ has prepared a fibrous adsorbent (PP-AM-PEI) through grafting AM onto PP fibers, followed by reaction with PEI. Though PP-AM-PEI had a similar adsorption capacity with PP-AM-HBP-NH_2_ adsorption fibers, its amino utilization efficiency (56.0%) was much lower than that of PP-AM-HBP-NH_2_, further highlighting the superiority of PP-AM-HBP-NH_2_ adsorbent.

#### Regeneration performance of PP-AM-HBP-NH_2_

**Figure 11:**
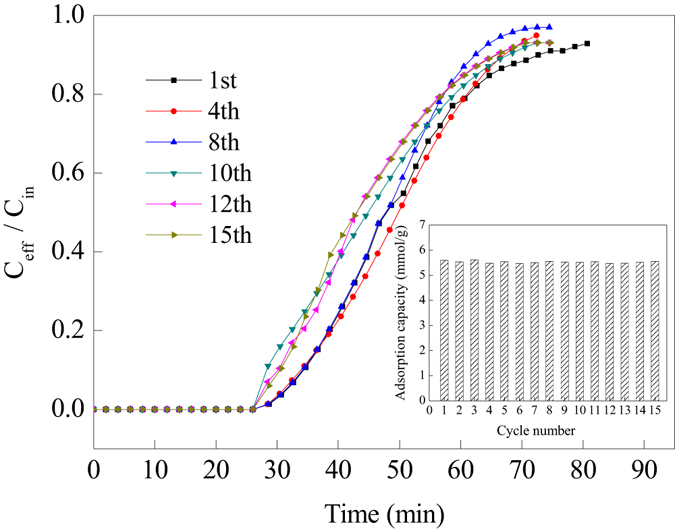
Breakthrough curves of CO_2_ adsorption on fresh and regenerated adsorbents (alkyl amino content was 6.39 mmol/g) and the CO_2_ adsorption capacities (inset).

The PP-AM-HBP-NH_2_ adsorbent was processed for 15 cycles of adsorption (at 25 °C) -desorption (at 90 °C), and the results were shown in Fig. 11. After 15 cycles, no significant changes in CO_2_ adsorption capacities were observed, and the adsorption capacity of regenerated PP-AM-HBP-NH_2_ remained at 5.60 ± 0.1 mmol/g (Fig. 11 inset). It was evident that the hyperbranch-structured adsorbent could remain stability after multiple regeneration cycles and maintain its adsorption capacity for CO_2_.

## Conclusions

A fibrous adsorbent with amino-terminated hyperbranch structure (PP-AM-HBP-NH_2_) could be conveniently prepared by grafting hyperbranch oligomers HBP-NH_2_ onto acrylamide modified polypropylene fibers. This study showed that the PP-AM-HBP-NH_2_ fibers could effectively adsorb CO_2_ due to its high amino density and hyperbranch structure, the maximum CO_2_ adsorption capacity and amino utilization efficiency of PP-AM-HBP-NH_2_ could reach 5.64 mmol/g and 88.2%, respectively. The hyperbranched structure as well as the good swelling properties of the grafted HBP-NH_2_ could provide more active sites for CO_2_ adsorption and reduced the mass transfer resistance to CO_2_, thus remarkably enhance the adsorption ability and amino utilization efficiency of the adsorbent. The prepared PP-AM-HBP-NH_2_ adsorbent also showed excellent regeneration stability, it could maintaine almost the same CO_2_ adsorption capacity after 15 cycles of adsorption-desorption.

## Experimental Section

### Materials and reagents

All reagents were purchased as analytical grade (AR) and used without further purification. Polypropylene (PP) fibers were provided by Xinshun Special Fiber Company (Zhongshan, China). Pentaethylenehexamine (PEHA) was purchased from Guangzhou Reagent Company. Acrylamide (AM), ethanol and ammonium ferrous sulfate [(NH_4_)_2_SO_4_·FeSO_4_·6H_2_O)] were purchased from Tianjin Fuchen Chemical Reagents Factory.

### Preparation of hyperbranch-structured fibers

#### Synthesis of HBP-NH_2_

The preparation process of HBP-NH_2_ was illustrated in Fig. 1(a). In a typical process, 0.5 mol PEHA was dissolved in 50 mL anhydrous methanol at 0 °C under N_2_ atmosphere. 0.5 mol MA dissolved in 50 mL anhydrous methanol was added dropwise to the above PEHA solution. PEHA reacted with MA through Michael addition reaction to produce a light yellow liquid intermediate (addition product). The intermediate was transferred to a round bottom flask in a rotary evaporation apparatus, and heated at 60 °C for 1 h to remove methanol solvent; then it was kept at 100 °C for 2 h for condensation reaction. After that, the reaction temperature was elevated to 140 °C and kept for 2 h for the completion of the self-condensation. The generated methanol in the reaction was removed by rotary evaporation in vacuum. The obtained amino-terminated hyperbranched polyamine was denominated as HBP-NH_2_.

#### Synthesis of PP-AM-HBP-NH_2_

The preparation process of PP-AM-HBP-NH_2_ was illustrated in Fig. 1(b). In a typical synthesis procedure: firstly, PP was subjected to γ-ray irradiation at a dosage rate of 0.837 kGy/h to obtain preirradiated PP fibers. Then AM was grafted onto PP fibers by the following procedure^10^: 85 g H_2_O and 0.08 g (NH_4_)_2_SO_4_·FeSO_4_·6H_2_O were put in a 100 mL three-necked flask, and oxygen in the solution was removed by purging nitrogen for 30 min, then 1.00 g of PP fibers and 15.00 g of AM were put into the flask and the grafting reaction lasted for 2 h at 70 °C. After the grafting step, the fibers were washed with boiling deionized water for several times to completely remove residual monomers and homopolymers, and the AM-grafted fibers PP-AM was dried in vacuum at 60 °C for 24 h^24–26^. Afterwards, HBP-NH_2_ was introduced onto PP-AM fibers by reacting with HBP-NH_2_ (20–90 wt% aqueous solution) for 6 h. The obtained fibers PP-AM-HBP-NH_2_ was rinsed with deionized water and ethanol, and dried at 60 °C for 24 h. The grafting degree of PP-AM (G_AM_, %) was calculated by the following Eq. (1):

1 $${G}_{AM}=\frac{{W}_{2}-{W}_{1}}{{W}_{1}}\times 100 \% $$

where *W*_*1*_ and *W*_*2*_ are the weights (g) of PP and PP-AM, respectively.

Alkyl amino content (n, mmol/g) of PP-AM-HBP-NH_2_ (amide excluded) was calculated by Eq. (2):

2 $$n=\frac{{{\rm{n}}}_{{\rm{2}}}{W}_{3}-{n}_{1}{W}_{2}{n}_{0}({W}_{3}-{W}_{2})}{{W}_{3}}$$

where *n*_*0*_ and *n*_*1*_ are the amide content (mmol/g) of HBP-NH_2_ and PP-AM, respectively, *n*_*2*_ is the amino content (mmol/g) of PP-AM-HBP-NH_2_, *W*_*3*_ is the weight (g) of PP-AM-HBP-NH_2_.

Since the preparation of HBP-NH_2_ is multi-step and the reaction sites are non-unique, structures of products could be various. Based on the preparation principle of PP-AM-HBP-NH_2_, a possible structure of PP-AM-HBP-NH_2_ is illustrated in Fig. 2.

#### Physical and chemical characterization

Elemental analysis (Elementar, Vario EL), Infrared (IR) spectra (Tensor-27 spectrometer) and X-ray photoelectron spectroscopy (ESCALAB 250, Thermo-VG Scientific) were employed to determine the composition and chemical structures of different samples, which were extracted by using ethanol^24, 27^. High performance liquid chromatograph (Waters 600), and gel column (TSK-GEL G2000 SW XL, 7.8 mm × 300 mm) were employed to determine the molecular weight of HBP-NH_2_NH_2_. Test conditions: mobile phase was phosphate buffer (pH = 7.0); detection wavelength was set at 222 nm; flow rate was 0.5 mL/min; column temperature was 300 °C; injection volume was 10 uL.

Ultra-depth three-dimensional microscope (VHX-1000C) was employed to observe the morphology and measure the diameter of fibers. The swelling degree (Sd, %) was calculated as follows:

3 $$Sd=\frac{{D}_{2}-{D}_{1}}{{D}_{1}}\times 100 \% $$

where *D*_*1*_ and *D*_*2*_ are the diameter of fibers in dry conditions and after swelling in water at 25 °C, (μm), respectively.

#### CO_2_ adsorption experiment

Breakthrough curves were used to characterize the CO_2_ adsorption performances of all samples in the presence of water. 1.00 g adsorbent sample was tightly packed in an glass column (*Φ* = 1.3 cm), into which a dry nitrogen flow was introduced at a flow rate of 30 mL min^−1^ for 0.5 h to remove the air and excess water in the column. Then, the dry CO_2_/N_2_ mixed gas (CO_2_: N_2_ = 1:9 (volume ratio)) was introduced through the column at a flow rate of 30 mL/min. The inlet/outlet concentrations of CO_2_ were analyzed every two minutes, using a Techcomp 7900 gas chromatograph equipped with a thermal-conductivity detector (TCD). The effect of adsorption temperature on the adsorption was investigated in the range of 10 to 80 °C. After adsorption, pure nitrogen gas at a flow rate of 30 mL/min was introduced through the tube at 90 °C to regenerate the spent adsorbent sample.

The adsorption capacity was calculated as follows:

4 $$Q={\int }_{0}^{{\rm{t}}}({C}_{{\rm{in}}}-{C}_{{\rm{eff}}})V\mathrm{dt}/22.4W$$

where *Q* is the adsorption capacity (mmol CO_2_/g); *t* is the adsorption time (min); *C*_*in*_ and *C*_*eff*_ are the influent and effluent concentrations of CO_2_ (vol%), respectively; *V* is the total flow rate, 30 mL/min; *W* and the constant (22.4) are the weight of sample (g) and molar volume of gas (mL/mmol), respectively.

## Electronic supplementary material

Supplementary PDF File

## References

[1] Lee SY, Park SJ (2015). A Review on Solid Adsorbents for Carbon Dioxide Capture. J. Ind. Eng. Chem..

[2] Jassim MS (2007). Carbon Dioxide Absorption and Desorption in Aqueous Monoethanolamino Solutions in A Rotating Packed Bed. Ind. Eng. Chem. Res..

[3] Loganathan S (2014). CO_2_ Adsorption Kinetics on Mesoporous Silica under Wide Range of Pressure and Temperature. Chem. Eng. J.

[4] Patil U (2012). Silicon Oxynitrides of KCC-1, SBA-15 and MCM-41 for CO_2_ Capture with Excellent Stability and Regenerability. Chem. Sci.

[5] Yoo CJ (2015). Probing Intramolecular versus Intermolecular CO_2_ Adsorption on Amine-Grafted SBA-15. Langmuir.

[6] Kamimura Y, Endo A (2016). CO_2_ Adsorption-Desorption Performance of Mesoporous Zirconium Hydroxide with Robust Water Durability. Phys. Chem. Chem. Phys..

[7] Bezerra DP (2011). Adsorption of CO_2_ on Nitrogen-enriched Activated Carbon and Zeolite 13X. Adsorption.

[8] Alabadi A (2016). Imine-Linked Polymer Based Nitrogen-Doped Porous Activated Carbon for Efficient and Selective CO_2_ Capture. Sci. Rep.

[9] Pal A (2016). Experimental Investigation of CO_2_ Adsorption onto A Carbon Based Consolidated Composite Adsorbent for Adsorption Cooling Application. Appl. Therm. Eng..

[10] Wu QH (2014). Effect of Surface Chemistry of Polyethyleneimine-grafted Polypropylene Fiber on Its CO_2_ Adsorption. RSC Adv.

[11] Li PY (2008). CO_2_ Capture by Polyethylenimine-Modified Fibrous Adsorbent. Langmuir.

[12] Yang Y (2010). Preparation and Characterization of A Solid Amine Adsorbent for Capturing CO_2_ by Grafting Allylamino onto PAN Fiber. Langmuir.

[13] Zhuang LZ (2013). Preparation of A Solid Amine Adsorbent Based on Polypropylene Fiber and Its Performance for CO_2_ Capture. J. Mater. Res..

[14] Lin RJ (2013). Design of A Viscose Based Solid Amine Fiber: Effect of Its Chemical Structure on Adsorption Properties for Carbon Dioxide. J. Colloid. Interf. Sci..

[15] Xu T (2015). Preparation of Polypropylene Based Hyperbranched Absorbent Fibers and The Study of Their Adsorption of CO_2_. RSC Adv..

[16] Gao C, Yan D (2004). Hyperbranched Polyamines: from Synthesis to Applications. Prog. Polym. Sci..

[17] Xu L, Ye Z (2013). A Pd-diimine Catalytic Inimer for Synthesis of Polyethylenes of Hyperbranched-on-hyperbranched and Star Architectures. Chem. Commun..

[18] Schubert C (2016). Can Hyperbranched Polymers Entangle? Effect of Hydrogen Bonding on Entanglement Transition and Thermorheological Properties of Hyperbranched Polyglycerol Melts. Macromolecules.

[19] Chen YS (2015). Synthesis and Application of Polyethylene-Based Functionalized Hyperbranched polyamines. Prog. Polym. Sci..

[20] Hu WZ (2015). Hyper-Branched Polymer Grafting Graphene Oxide as An Effective Flame Retardant and Smoke Suppressant for Polystyrene. J. Hazard. Mater..

[21] Chen QJ (2010). Role of Pore Structure of Activated Carbon Fibers in The Catalytic Oxidation of H_2_S. Ind. Eng. Chem. Res..

[22] Fischer W (2010). Hyperbranched Polyamines for Transfection. Top. Curr. Chem.

[23] Morales-Lara F (2016). Grafting The Surface of Carbon Nanotubes and Carbon Black with The Chemical Properties of Hyperbranched Polyamines. Sci. Technol. Adv. Mat..

[24] Thakur VK (2014). Graft Copolymers of Natural Fibers for Green Composites. Carbohyd. Polym..

[25] Zhang KK (2016). Improve The Flame Retardancy of Cellulose Fibers by Grafting Zinc Ion. Carbohyd. Polym..

[26] Su SZ (2017). Enhancing Adsorption of U(VI) onto EDTA Modified L. Cylindrica Using Epichlorohydrin and Ethylenediamine as A Bridge. Sci. Rep.

[27] Hansson S (2013). Grafting Efficiency of Synthetic Polymers onto Biomaterials: A Comparative Study of Grafting-from Versus Grafting-to. Biomacromolecules.

