# Supplementary material for: Preparation and Properties of A Hyperbranch-Structured Polyamine adsorbent for Carbon Dioxide Capture
Source: Sci Rep. 2017 Jun 20;7:3913. doi: 10.1038/s41598-017-04329-w (PMC5478640; doi:10.1038/s41598-017-04329-w)
Supplement: Supplementary file 1 — Supplementary PDF File [file 41598_2017_4329_MOESM1_ESM.pdf]

Supporting Information

Preparation and Properties of A Hyperbranch-Structured Polyamine adsorbent for Carbon Dioxide Capture

Hui He [a,c], Yajie Hu [a], Shuixia Chen [a,b*], Linzhou Zhuang [a], Beibei Ma [a], Qinghua Wu [a]

a. PCFM Lab, School of Chemistry, Sun Yat-Sen University, Guangzhou 510275, PR China

b. Materials Science Institute, Sun Yat-Sen University, Guangzhou 510275, PR China

c. College of Light Industry and Food Engineering, Guangxi University, Nanning 530004, PR China

Amino content of PP-AM-HBP-NH_2 adsorbent

As showed by the elemental analysis results, no nitrogen and oxygen was detected in PP fibers, but certain amount of nitrogen and oxygen were measured in the PP-AM-HBP-NH_2 fibers due to the introduction of AM and HBP-NH_2 to the PP fibers during the grafting process. Thus, the nitrogen contents (wt%) and oxygen contents (wt%) could be used to estimate the amount of N and O introduced. As we know, only amide group contains oxygen, thus the amide content (n_1', mmol/g) of PP-AM-HBP-NH_2 could be calculated from the oxygen mole content (that is, amide content in mmol/g of PP-AM-HBP-NH_2 is equal to the oxygen mole content). The alkyl amino content (n', mmol/g) of PP-AM-HBP-NH_2 (amide excluded) was calculated by equation (S1):

$$n' = n_2' - n_1' \qquad \text{(S1)}$$

* Corresponding author. *E-mail address*: cescsx@mail.sysu.edu.cn;

where n_2' is the total amino content (mmol/g) of PP-AM-HBP-NH_2.

Amine titration was used to measure the amino content of PP-AM-HBP-NH_2 fibers. Twenty milligrams of each fiber samples were added to 10 ml HCl (0.05 M). The mixture was shaken for 30 min in a sealed container under N_2 atmosphere and then filtered to remove fiber samples from the solution. Immediately after filtration, the solution was titrated with NaOH (0.05 M) as a titrant with a seal of parafilm that was maintained around both the electrode and the burette, in order to avoid probable HCl evaporation and so change in acid concentration. The PP-AM contains no alkyl amino calculated from amine titration due to only alkyl amino (amide excluded) can react with HCl during the titration process.

Table S1 The amino content of PP-AM-HBP-NH_2

N (wt%)	O (wt%)	Amino amount (mmol/g)	Amide content (mmol/g)	Alkyl amino content (mmol/g)		
				calculated from equation (S1)	calculated from equation (2)	calculated from amine titration
15.36	9.32	10.97	5.82	5.15	5.19	5.12
16.09	9.38	11.49	5.86	5.63	5.71	5.59
16.82	9.68	12.01	6.05	5.96	6.04	5.97
17.56	9.98	12.54	6.23	6.31	6.39	6.35
18.22	11.61	13.01	7.25	5.76	5.84	5.79
18.20	12.15	13.00	7.59	5.41	5.46	5.35

The alkyl amino content of PP-AM-HBP-NH_2 calculated from equation (2) in the manuscript (based on the weight gain after grafting) and that calculated from equation (S1) and amine titration are very close (Table S1). We believe that the amino or alkyl amino contents of samples calculated from EA are reliable.

Indeed, XPS has been employed to analyze the surface chemical properties of PP-AM-HBP-NH_2 (Figure 6 in the manuscript). Its N 1s peak could be resolved into four peaks at 400.10 eV (13.56%), 399.25 eV (10.07%), 398.65 eV (24.11%), and 398.07 eV (52.26%), which were the characteristics of amide, tertiary amino, primary amino, and secondary amino, respectively. Meanwhile, the absorption peaks of FT-IR spectra at 1573 cm^{-1}, 1460 cm^{-1}, and 1116 cm^{-1} are corresponding to the bending of amino groups (N-H) in PP-AM-HBP-NH_2 (Figure 5). The presence of amide, tertiary amino, primary amino, and secondary amino indicated that the HBP-NH_2 was grafted onto the PP-AM, and the target hyperbranch-structured fibers were successfully prepared.

Table S2 The nitrogen content of PP-AM-HBP-NH_2

N content measured by EA (wt%)	15.36	16.82	17.56	18.22
N content measured by XPS (wt%)	14.47	15.91	16.69	17.43

The nitrogen content of PP-AM-HBP-NH_2 measured by EA and that measured by XPS are very close (Table S2), which further indicated that the alkyl amino contents of samples calculated from EA are reliable.

The BET surface area characterization

The surface area of the PP fibers calculated from the dimensions was 0.0815 m^2/g. Actually the BET surface area of PP and PP-AM-HBP-NH_2 fibers has also been measured from N_2 adsorption-desorption isotherms at 77.35 K, which were 4.08 m^2/g and 5.82 m^2/g, respectively (Table S3). The BET surface area of PP fibers did not change significantly after grafting HBP-NH_2. Compared with BET surface area of PP fibers, the surface area of the PP fibers calculated from the dimensions was lower than

it, which indicated that a large number of pores exist within the PP fibers.

According to your comment, the BET surface area of PP and PP-AM-HBP-NH_2 fibers has been supplied in the supporting information.

Table S3 The BET surface area and pore volume of PP and PP-AM-HBP-NH_2

Materials	BET surface area (m^2/g)	Pore volume (cm^3/g)
PP	4.08	0.01
PP-AM-HBP-NH_2	5.82	0.01

Ultra-depth three-dimensional microscope images

A fibrous adsorbent with amino-terminated hyperbranched structure (PP-AM-HBP-NH_2) was prepared by grafting hyperbranched polyamine (HBP-NH_2) onto the acrylamide-modified polypropylene fibres (PP-AM). During the grafting of AM onto PP fibers, AM copolymerized with PP to form PP-AM copolymers with polyamide oligomer as side chains, which provided abundant active sites for introducing HBP-NH_2 onto the PP fibers. The diameter of the PP-AM (the grafting degree was 320%) thus was increased to 95.52 μm. And then its diameter further increased to 116 um after grafting HBP-NH_2 onto PP-AM (Figure S1). Meanwhile, the nitrogen content of PP-AM-HBP-NH_2 has an increment of 6.31 wt% from 12.14 wt% (PP-AM) to 18.45 wt% (Figure 4).

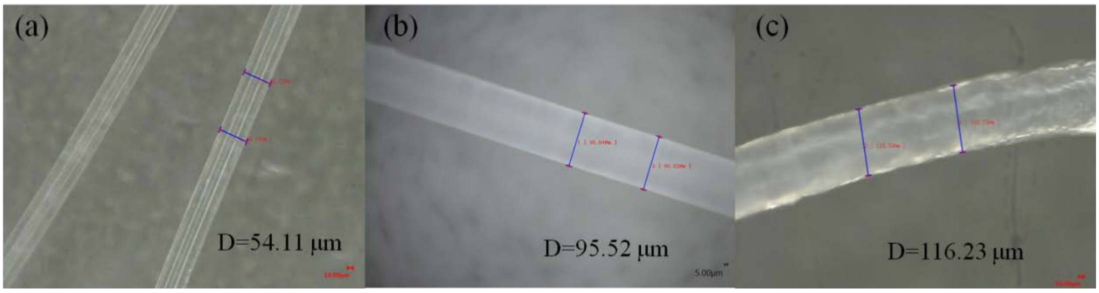

Figure S1 Ultra-depth three-dimensional microscope images of PP (a), PP-AM (b), and PP-AM-HBP-NH_2 (c) under dry conditions

Comparison of CO_2 adsorption capacity of amine functionalized fibrous

Compared with PEI modified fibrous adsorbents (PP-AM-PEI[10], PP-GMA-PEI[10], GF-ECH-PEI[11], VF-PEI[S1]) reported (Table S4), PP-AM-HBP-NH_2 adsorption fibers showed relatively high adsorption efficiency. More interestingly, Wu[10] has prepared a fibrous adsorbent (PP-AM-PEI) through grafting AM onto PP fibers, followed by reaction with PEI. Though PP-AM-PEI had a similar adsorption capacity with PP-AM-HBP-NH_2 adsorption fibers, its amino utilization efficiency (56.0 %) was much lower than that of PP-AM-HBP-NH_2, further highlighting the superiority of PP-AM-HBP-NH_2 adsorbent.

Table S4 Comparison of CO_2 adsorption capacity of PEI and HBP-NH_2 functionalized fibrous sorbents

Substrate*	Grafted monomer**	Amine	Adsorption capacity (mmol/g)	Amino utilization efficiency (%)	Ref.
PP	AM	HBP-NH_2	5.64	88.2	This work
PP	AM	PEI	5.91	56.0	10
PP	GMA	PEI	2.51	26.9	10
GF	ECH	PEI	4.12	57.0	11
VF		PEI	4.11	29.38	S1

* GF: glass fibers, VF: viscose fibers, ** ECH: epichlorohydrin, GMA: glycidyl methacrylate. Adsorption temperature: 25 ^{o}C, CO_2 concentration: 10%.

REFERENCES

S1 He. H. et al. The Oxidation of Viscose Fiber Optimized by Response Surface Methodology and Its Further Amination with PEI for CO_2 Adsorption. *Cellulose* **23**, 2539-2548 (2016).
